# Considering the effect of Pi rebinding on myosin dynamics based on the distinct functions of cardiac and skeletal myosin

**DOI:** 10.3389/fphys.2025.1605321

**Published:** 2025-05-16

**Authors:** Motoshi Kaya

**Affiliations:** Department of Physics, Graduate School of Science, University of Tokyo, Bunkyo, Japan

**Keywords:** cardiac myosin, skeletal myosin, conformational dynamics, power stroke reversal, functional adaptation

## Abstract

In recent years, significant advances have been made in various fields related to the study of myosin, including muscle fiber research, structural studies, and single-molecule measurements. These advances have provided detailed insights into the chemical and mechanical dynamics of myosin. As a result, numerous studies have been conducted on the temporal relationships between phosphate (Pi) release and force generation via myosin structural changes. These structural changes are a critical step in the myosin mechanochemical cycle. Two models have been proposed to explain this process. The first model proposes that force is generated by power stroke after Pi release, while the second model proposes that Pi is released after force generation by the power stroke. Furthermore, a comprehensive model has been proposed to elucidate phenomena predicted by both models. In this article, we explore the structural changes in myosin associated with Pi binding from a different perspective, based on our research, suggesting that the molecular structural dynamics associated with Pi rebinding, as well as its impact on force generation in molecular ensembles, exhibit distinct characteristics between cardiac myosin and skeletal myosin. It is widely acknowledged that an excessive increase in Pi concentration can adversely affect contraction function and contribute to the development of contraction-related diseases in both cardiac and skeletal muscle. In response to such adverse conditions, cardiac and skeletal myosin may employ different mechanisms to counteract the reduction in contractile capacity. Examining these potential mechanisms could facilitate the development of novel therapeutic strategies.

## Introduction

Myosin is a motor enzyme that converts the chemical energy derived from ATP hydrolysis into mechanical work, thereby generating the driving force for muscle and cardiac contraction. It is imperative to elucidate the extent to which the free energy from ATP hydrolysis is utilized by a single myosin molecule to drive contraction. Since the development of single-molecule protein measurements using optical tweezers, the measurement of the forces and displacements of single skeletal myosin molecules has constituted a primary area of research focus. Numerous studies have debated key parameters such as power stroke size and stall force (Finer et al., 1994; Ishijima et al., 1994; Molloy et al., 1995; Kitamura et al., 1999; Capitanio et al., 2006; Takagi et al., 2006; Kaya and Higuchi, 2010). In recent years, significant advances have been made in single-molecule measurement techniques, leading to substantial improvements in spatiotemporal resolution (Capitanio et al., 2012; Woody et al., 2019; Hwang et al., 2021). Furthermore, the development of high-speed atomic force microscopy (AFM) (Fujita et al., 2019; Moretto et al., 2022) has enabled direct visualization of myosin molecular dynamics, allowing for more detailed discussions of its mechanochemical dynamics. These technological advances have facilitated the elucidation of the temporal relationship between Pi/ADP release within the ATPase cycle and the transition states between weak and strong binding of myosin—topics that have been debated for decades. The primary discussion centers on whether Pi release occurs before or after the structural change of myosin (power stroke). In the field of skeletal myosin research, various experimental approaches—including muscle fiber studies, solution-based FRET experiments, motility assays, and optical tweezer measurements—have contributed to the mechanical analysis in response to Pi concentration changes. These studies have led to the proposal of two models: the Pi release-first model, where Pi release occurs before the power stroke (Pate and Cooke, 1989; Smith, 2014; Llinas et al., 2015), and the power stroke-first model, where Pi is released after the power stroke (Dantzig et al., 1992; Tesi et al., 2002; Muretta et al., 2015; Woody et al., 2019; Governali et al., 2020; Scott et al., 2021). Recent single-molecule optical tweezer studies on cardiac myosin (Woody et al., 2019) and myosin V (Scott et al., 2021) suggest that their mechanical responses to Pi concentration are consistent with the power stroke-first model. For detailed discussions of models for Pi release and myosin structural changes, we refer readers to extensive review articles (Debold, 2021; Mansson et al., 2023).

Woody et al. (2019) demonstrated that a single cardiac myosin molecule frequently undergoes power stroke reversal under applied load and that this tendency is further enhanced by Pi rebinding. In contrast, Scott et al. (2021) reported that myosin V did not exhibit a significant Pi concentration-dependent power stroke reversal under loads presumably below 1 pN in their direct observations. Hence, a further intriguing observation from these studies is that the structural changes in myosin associated with Pi rebinding may be isoform-specific, reflecting functional adaptations among different classes of myosin. According to the power stroke-first model proposed by Woody et al. (2019), an increased frequency of Pi concentration-dependent power stroke reversal would be expected to reduce isometric contraction force, slow contraction velocity, and decrease ATPase rate. However, several experimental observations on skeletal myosin/muscle challenge this explanation: the minimal change in muscle fiber contraction velocity upon Pi elevation (Pate and Cooke, 1989), the increase in actin sliding velocity in motility assays at low pH at high Pi concentrations (Debold et al., 2011), and the relatively unchanged ATPase rate (Linari et al., 2010). These findings imply that skeletal myosin rarely undergoes power stroke reversal. Instead, it is hypothesized that they likely follow an alternative reaction pathway in which they dissociate from the post-power stroke state (i.e., A.M.ADP) via Pi rebinding (Linari et al., 2010; Scott et al., 2021).

## Dynamics of single cardiac myosin differ from single fast skeletal myosin in response to load

**FIGURE 1:**
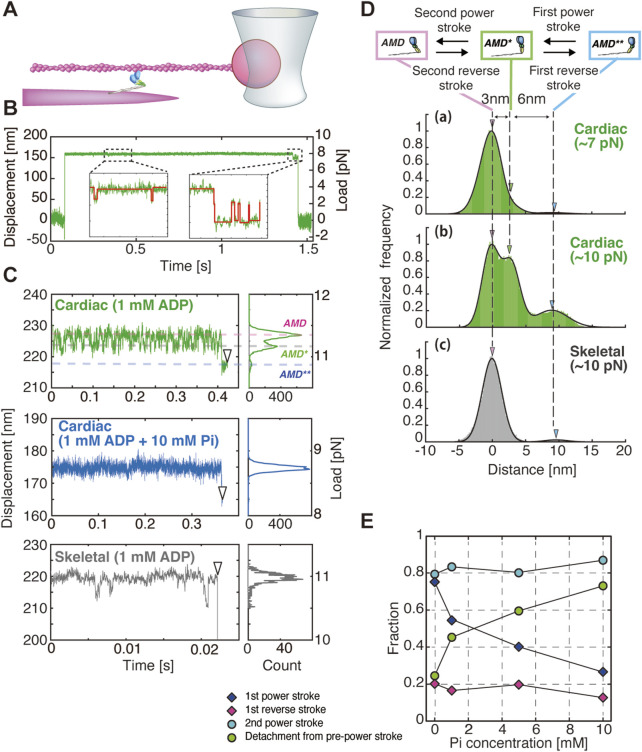
Single-molecule measurements of myosin dynamics using an optical tweezer system. **(A–D)** were modified from Hwang et al. (2021). **(A)** An actin-bead complex was interacted with a single myosin molecule embedded in a myosin filament, and bead displacement was measured to evaluate the mechanical response of the myosin molecule. Gelsolin-conjugated beads were attached to the barbed ends of actin filaments to ensure the application of hindering loads by displacing the beads away from the fluorescently labeled myofilaments. **(B)** Example of bead displacement traces for a single cardiac myosin molecule. (Inset) Enlarged view of the displacement trace in the dashed area, with steps detected using the vbFRet algorithm (red lines) (Bronson et al., 2009). The laser is displaced by approximately 150 nm. With a trap stiffness of 0.05 pN/nm, this applies ∼8 pN of force, likely stretching the myosin by 2–3 nm. **(C)** Examples of bead displacement traces and histograms for cardiac and skeletal myosin. **(D)** Cumulative histograms of bead displacement traces for cardiac and skeletal myosin at 1 mM ADP: **(a)** cardiac myosin at 6–8.5 pN (n = 9), **(b)** cardiac myosin at 8.5–12 pN (n = 8), and **(c)** skeletal myosin at 8.5–12 pN (n = 15). The corresponding transition states—pre-power stroke state (AMD**), first power stroke state (AMD*), and second power stroke state (AMD)—are shown in the upper diagram. White arrowheads indicate detachment points. **(E)** Relationship between the fraction of each structural event (power stroke, power stroke reversal and detachment) and Pi concentration.

The amino acid sequence identity between cardiac myosin and skeletal myosin is extremely high, ranging from 80% to 90% (Lee et al., 2019). In particular, given the identical gene for cardiac myosin and slow-twitch myosin heavy chain (MYH7), it can be expected that both cardiac myosin and skeletal myosin possess highly similar molecular properties. However, it is not inconceivable to hypothesize that their molecular properties may differ, given that skeletal myosin is primarily responsible for muscle contraction requiring high contractile force and velocity, whereas cardiac myosin is involved in heart contraction, which necessitates the maintenance of stable systolic blood pressure over a prolonged period. Therefore, we analyzed the characteristics of β-cardiac myosin purified from porcine ventricles and fast-twitch skeletal myosin purified from the rabbit psoas muscle by measuring the structural changes of single myosin molecules under load and by assessing force generation in multimolecular filaments composed of approximately 20 molecules (Hwang et al., 2021). In this study, the number of myosin molecules interacting with actin could be controlled by adjusting the mixing ratio of wild-type myosin and myosin rods (myosin molecules with the truncated heads). In an assay with a molar ratio of [1:200], 0–1 myosin molecules could interact with actin, whereas in an assay with a molar ratio of [1:2], up to 20 molecules could interact with actin (Kaya et al., 2017; Hwang et al., 2021). In the optical tweezers experiment, beads attached to actin were trapped using optical tweezers, and the actin was brought near the myosin filament to facilitate interaction with myosin. This setup allowed the measurement of force generation and structural changes in myosin molecules. For experiments measuring the structural changes of a single myosin molecule, measurements were conducted in a solution containing 1 mM ADP and 0–10 mM Pi (Figure 1A). During the course of the actin-myosin interactions, a hindering force of 6–17 pN was applied to myosin by pulling the actin-bead complex. The structural changes in myosin in response to this load were analyzed based on the bead movement (Figure 1B). The main finding of this study is that the displacement distribution of beads in myosin exhibited three distinct peaks, with distances of 6 nm and 3 nm between these peaks (Figures 1C, D). This observation was consistent with the two-step power stroke sizes estimated from force measurements of myofilaments (Hwang et al., 2021). Force generation accompanied by a two-step power stroke has been observed in myosin I (Veigel et al., 1999), myosin V (Veigel et al., 2005), and skeletal myosin (Capitanio et al., 2006; Kaya et al., 2017); however, this was the first report of such behavior in cardiac myosin.

One may argue that these back-and-forth displacements of a single myosin molecule could potentially arise from detachment and reattachment of one of its heads. If this were the case, the magnitude of the backward displacement would be expected to increase proportionally with the additional load applied to the bound head. However, this expectation is not supported by our experimental observations, which argues against this mechanism. Therefore, even at the single-molecule level of cardiac myosin in the absence of ATP, structural changes corresponding to the two-step power stroke were observed. Under the condition of 1 mM ADP, it was found that both cardiac myosin and skeletal myosin entered the second post-power stroke (AMD) state, which precedes ATP binding, immediately after the start of the measurement (Figure 1C). The applied load on myosin resulted in cardiac myosin undergoing power strokes and power stroke reversals multiple times, transitioning between the second post-power stroke state (AMD), the first post-power stroke state (AMD), and the pre-power stroke state (AMD**). Ultimately, 92% (24/26) of the myosin molecules dissociated from the pre-power stroke state (Figure 1C). In contrast, skeletal myosin demonstrated minimal structural changes associated with power strokes and power stroke reversals, remaining predominantly in the second post-power stroke state for an extended period. Subsequently, 83% (29/35) of the myosin molecules dissociated directly from this state (Figure 1C). These findings imply that cardiac myosin is capable of undergoing power strokes and reversals even in the absence of Pi binding. In contrast, skeletal myosin, once it reaches the post-power stroke state, remains in that state with minimal structural change.

## Force generation in cardiac and skeletal myosin ensembles reflects the properties of individual myosin molecules

**FIGURE 2:**
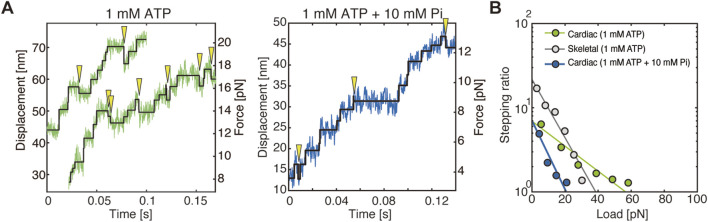
Step analysis in force measurements of myofilaments. Figures were modified from Hwang et al. (2021). **(A)** Example of stepwise actin displacement traces generated by cardiac myofilaments, with detected steps indicated by black lines. Yellow arrowheads indicate observed backward steps. **(B)** Relationship between the stepping ratio and applied load for cardiac and skeletal myofilaments. The abscissa of the linear fitting curves indicates the load at which the stepping ratio equals 1, representing the stall force.

A comparison of the force generation between cardiac myosin and skeletal myosin multi-molecule filaments, each prepared so that approximately 20 molecules could interact with actin, the cardiac myofilament exhibited a slower velocity as expected. However, the cardiac myofilament generated approximately twice the maximum force and frequently exhibited backward steps (Figure 2A). The stall force of the cardiac myofilament, estimated from the relationship between stepping ratio (the ratio between the number of forward and backward steps) and load, was approximately 2.5 times that of the skeletal myofilament (Figure 2B). These characteristics can be attributed to the frequent power stroke reversals as observed in single-molecule experiments and to backward steps resulting from dissociation from the pre-power stroke state (see more details discussed later). In contrast, skeletal myofilaments exhibited almost no backward steps until high loads were applied, maintaining a high stepping ratio. This result is consistent with the single-molecule experiments, where skeletal myosin rarely underwent power stroke reversal.

## Pi rebinding accelerates the rate of detachment of cardiac myosin, but does not alter the rate of the power stroke reversal

In the results of single-molecule structural dynamics of cardiac myosin under 1 mM ADP with 1, 5, and 10 mM Pi, a significant change compared to the 0 mM Pi condition was the substantial increase in the frequency of dissociation from actin in the pre-power stroke state. The findings reveal that, under conditions of 0 mM Pi, 22% of myosin molecules dissociated, while 78% proceeded to the first power stroke. Conversely, at 10 mM Pi, 78% dissociated, and only 22% underwent the first power stroke (Figure 1E). Notably, no substantial changes were observed in other structural transitions, such as first power stroke reversal and second power stroke/power stroke reversal. These observations indicate that Pi binding exerts a substantial influence on the mechanochemical reactions that occur in the pre-power stroke state. Furthermore, in force measurements of myofilaments under conditions of 1 mM ATP + 10 mM Pi, the stall force decreased from approximately 57 pN (observed under 1 mM ATP + 0 mM Pi) to around 20 pN. However, the stepping ratio under unloaded conditions remained unchanged at approximately 7 (Figure 2B). Integrating the results from myofilament experiments and single-molecule measurements, it can be concluded that Pi binding reduces the power stroke rate in the pre-power stroke state of cardiac myosin and increases its tendency to dissociate from actin, leading to a significant decrease in stall force (Figure 2B). Since our single-molecule measurements showed that the power stroke reversal rate is independent of Pi concentration, it is unlikely that Pi binding in the post-power stroke state accelerates power stroke reversal under high Pi concentrations. This idea is further substantiated by the findings derived from myofilaments, which demonstrated that the stepping ratio in unloaded conditions remained unaffected by Pi concentration (Figure 2B). Conversely, it should be noted that the accelerated decrease in the stepping ratio under high Pi concentrations and high loads (Figure 2B) can also be explained by the report of Woody et al. (2019), which demonstrated that power stroke reversal increases in a load-dependent manner in the presence of ATP and Pi. Overall, the results from our single-molecule assay and myofilament assay are consistent, suggesting that Pi rebinding in cardiac myosin predominantly occurs in the pre-power stroke state, suppresses force generation by the first power stroke, and promotes dissociation from actin, which aligns with the Pi-release first model.

A comparison of our single-molecule experiments with those of previous studies reveals two distinguishing features. First, our experiments were conducted in the absence of ATP while varying ADP and Pi concentrations. Second, myosin structural transitions were evaluated under conditions of two-to threefold higher mechanical load. These distinctions entail both advantages and limitations. Conducting measurements in the absence of ATP ensures that myosin exhibits high affinity for actin, thereby minimizing the likelihood of underestimating conformational changes associated with the power stroke and its reversal. Furthermore, embedding native double-headed myosin molecules into filaments enhances actin-binding affinity compared to single-headed S1 constructs. As a result, our experimental system is particularly well-suited for detecting actin displacements generated by myosin undergoing structural transitions under high-load conditions, while minimizing the likelihood of detachment from actin. Despite the findings of prior studies indicating that the power stroke size of single-headed S1 constructs approaches zero under high-load conditions (Capitanio et al., 2012), our experimental results successfully detected a two-step power stroke of 6 + 3 nm even under such load. Furthermore, in both single-molecule and multi-molecule filament assays, the utilization of fluorescently labeled actin filaments facilitates visual confirmation and deliberate alignment along the longitudinal axis of myosin, thereby ensuring the accurate application of load. This experimental configuration mitigates the risk of underestimating the amplitude of the power stroke (Tanaka et al., 1998). While these advantages are notable, it is also possible that measurements conducted in the absence of ATP may have contributed to differences from previous studies. Woody et al. (2019) reported that power stroke reversal was observed only in the presence of added Pi under ATP-present conditions. In contrast, our experiments revealed that power stroke reversal occurred irrespective of Pi concentration. This discrepancy may be attributable to the presence or absence of competitive binding of ATP and Pi, as proposed by Amrute-Nayak et al. (2008). In our single-molecule experiments, we employed 200-nm beads to achieve higher temporal resolution. However, noise analysis revealed that power stroke events could not be reliably detected unless forces exceeded 6 pN. This limitation impeded our ability to observe structural transitions under low-load conditions. In contrast, Woody et al. (2019) examined dynamics under loads ranging from 0 to 5 pN, and Capitanio et al. (2012) extended this range to 0–10 pN. These differences in the applied load range may also underlie the contrasting findings regarding the Pi-dependence of power stroke reversal. Furthermore, the power stroke rate obtained from our single-molecule experiments is approximately 100–300 s^−1^, an order of magnitude lower than the values reported by Woody et al. (2019). This discrepancy can be attributed to the methodological difference between the two studies. Rather than estimating values by exponentially fitting ensemble-averaged displacement traces as Woody et al. (2019) did, we used a step detection algorithm (Bronson et al., 2009) to identify individual steps from displacement data. However, due to the detection limit inherent to the algorithm, steps shorter than 1 ms could not be detected. Hence, the discrepancies in observed results and models (i.e., Pi release-first model vs. power stroke-first model) may be attributed to variations in detection capability, as well as experimental conditions and system setups (i.e., myofilament + single trap with ADP + Pi vs. heavy meromyosin on bead pedestal + dual trap with ATP + Pi).

As for skeletal myosin, unfortunately, we have not tested the effect of Pi binding on skeletal myosin. However, in the absence of Pi, in contrast to cardiac myosin, skeletal myosin exhibits minimal power stroke reversal, with the exception of the high load range (Figures 1C,D). It could be argued that the current approach lacks sufficient temporal resolution, potentially hindering the detection of rapid structural changes in skeletal myosin. However, even without clear detection of individual steps, structural changes should still be reflected in the distribution. The measured distribution showed no clear separation between three distinct distributions, with the one near the structural state after the second power stroke (AMD) being dominant (Figure 1D, gray), suggesting that power stroke reversal is unlikely. Additionally, our myofilament experiments demonstrated that the probability of backward steps is significantly lower for skeletal myofilaments compared to cardiac myofilaments (Figure 2B). These results are consistent with those of Scott et al. (2021), who did not observe power stroke reversal in myosin Va. Given these findings, it is difficult to imagine that Pi rebinding would accelerate power stroke reversal in skeletal myosin. Instead, it may be more reasonable to consider that Pi rebinding leads to either a reversal or a branched reaction in which myosin dissociates from the post-power stroke state, as proposed for skeletal myosin (Baker et al., 2002; Debold et al., 2013), myosin Va (Scott et al., 2021), and even muscle fiber experiments (Takagi et al., 2004; Linari et al., 2010).

## Functional response of myosin in heart and muscle

Our single-molecule and myofilament experiments have demonstrated that cardiac myosin frequently undergoes power stroke reversal, transitioning between the post-power stroke and pre-power stroke states. This phenomenon enables prolonged actin attachment, resulting in an augmented duty ratio and facilitating the maintenance of stable tension by multi-molecule ensembles over a designated period. This property is highly advantageous for sustaining stable blood pressure during the systolic phase of the cardiac cycle (Hwang et al., 2021). In contrast, skeletal myosin rarely undergoes power stroke reversal, which accelerates the actomyosin interaction cycle, enhancing both force and velocity to increase power output. This characteristic is particularly beneficial for fast-twitch muscles.

In the heart, an increase in Pi concentration accompanies cardiac dysfunction and leads to various impairments. For instance, in heart failure, reduced left ventricular function and myocardial hypertrophy due to cardiac remodeling can cause microvascular blood flow disturbances, leading to ischemic conditions where oxygen supply becomes insufficient (Camici et al., 2012; Pelliccia et al., 2022). Under such conditions, a decrease in oxygen supply has been shown to reduces mitochondrial ATP production capacity, thereby leading to impaired Pi metabolism and excessive Pi accumulation (Flood et al., 2023). In this energy-deprived state, excessive contraction further disrupts the balance between ATP demand and supply. However, the mechanism of Pi rebinding-induced power stroke reversal followed by detachment has the potential to reduce energy consumption associated with actomyosin interactions. Consequently, it functions as a protective mechanism that prevents excessive ATP consumption and contraction. A similar phenomenon occurs in skeletal muscle during fatigue, resulting in excessive accumulation of Pi, similar to ischemic conditions observed in the heart. In such conditions, skeletal myosin undergoes either a reversal (Baker et al., 2002) or a bifurcated reaction (Linari et al., 2010; Scott et al., 2021) upon Pi reattachment, dissociating from actin in the post-power stroke state prior to ATP binding, thereby preventing excessive ATP consumption. Moreover, the reduction in the number of myosin molecules in a strongly bound state for prolonged periods, drag is minimized, preventing excessive reductions in contraction velocity (Debold et al., 2013).

## Conclusion

In this perspective article, the differences in the mechanical responses of cardiac and skeletal myosin to load, as well as the distinct effects of Pi rebinding, have been discussed. The debate on whether Pi release precedes or follows force generation remains unresolved; however, with remarkable advances in single-molecule techniques and structural studies, a definitive answer may soon emerge. While elucidating the temporal relationship between Pi release and force generation is undoubtedly important, the key point we emphasize in this article is that even highly homologous myosins may exhibit distinct molecular dynamics upon Pi rebinding. This suggests that cardiac and skeletal muscle contraction disorders should not be treated as identical pathological conditions. Instead, considering them as distinct disease mechanisms may accelerate the development of more targeted and effective therapeutic strategies.

## Data Availability

The original contributions presented in the study are included in the article/supplementary material, further inquiries can be directed to the corresponding author.
